# Retraction: Effects of gallotannin-enriched extract of Galla Rhois on the activation of apoptosis, cell cycle arrest, and inhibition of migration ability in LLC1 cells and LLC1 tumors

**DOI:** 10.3389/pore.2024.1612042

**Published:** 2024-12-12

**Authors:** Pathology and Oncology Research Editorial Office

Following publication, concerns were raised regarding the integrity of the images in the published figures. Two panels in Figure 5A appear to have been duplicated.

**FIGURE 5:**
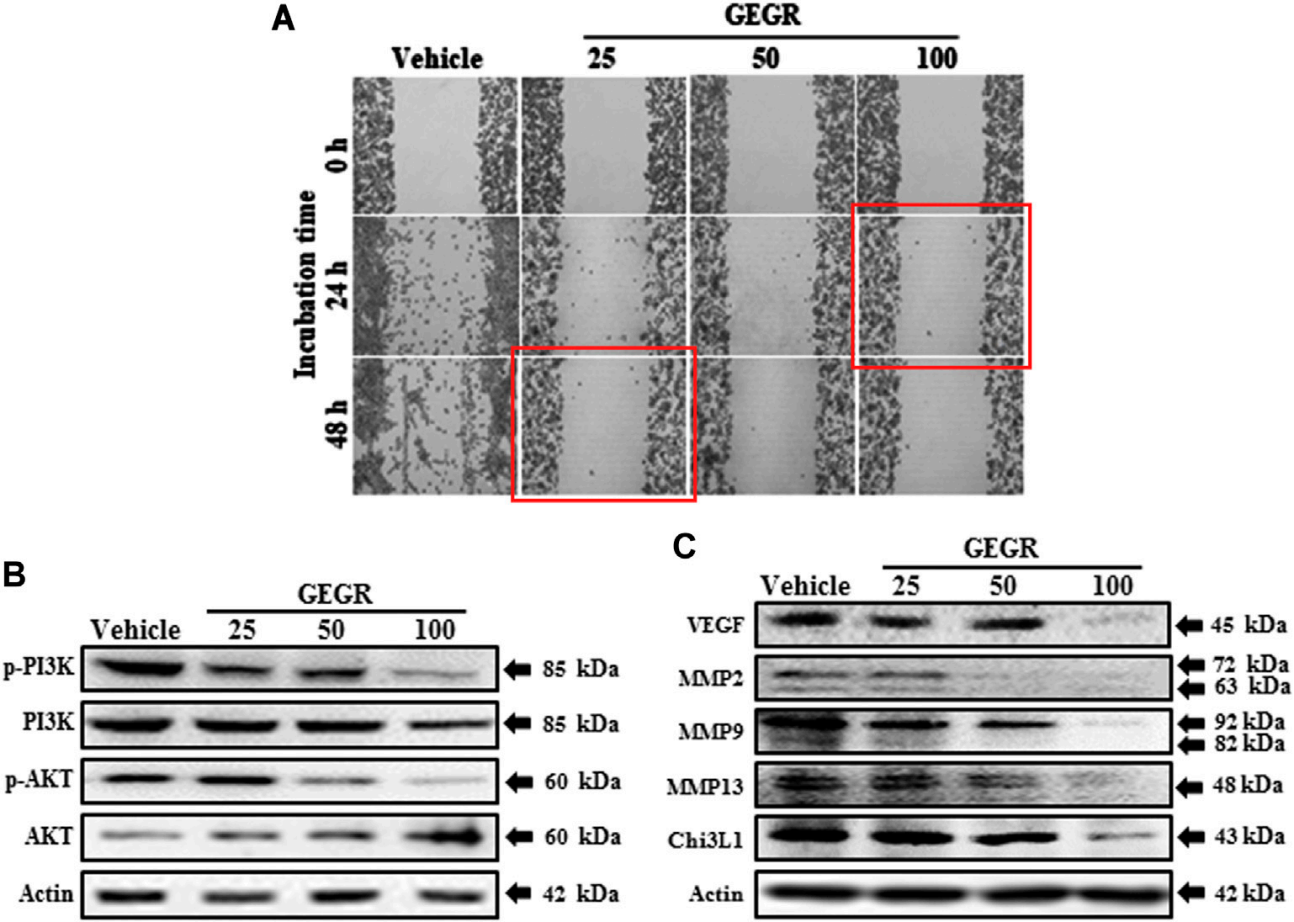
Analysis for cell migration in GEGR treated LLC1 cells. **(A)** The migration ability of LLC1 cells was analyzed by the wound healing assay, following treatment with 25, 50, and 100 μg/mL of GEGR. Morphological images of cells were captured after 24 h and 48 h of incubation at 200 × magnification. Two to three wells per group were used for preparing the artificial injury, and the closure rates were calculated in duplicate for each sample. **(B, C)** After treatment with 25, 50, and 100 μg/mL of GEGR for 24 h, the expression levels of nine proteins were determined using an imaging densitometer. The level of each protein was presented relative to the intensity of actin. Two to three dishes per group were used in the preparation of the total cell homogenates, and Western blot analysis was assayed in duplicate for each sample. Data are reported as the means ± SD. *, p < 0.05 compared to the Vehicle treated group. †, p < 0.05 compared to the 25GEGR treated group. §, p < 0.05 compared to the 50GEGR treated group.

The authors were contacted by the POR Editorial Office and asked to provide the complete raw data for their study. The authors failed to provide a satisfactory explanation during the investigation, which was conducted in accordance with POR policies. As a result, the data and conclusions of the article have been deemed unreliable and the article has been retracted.

This retraction was approved by the Editor-in-Chief of Pathology & Oncology Research.

The authors agree to this retraction. The communication has been recorded by the publisher.

